# Development of a Highly Reliable PbS QDs-Based SWIR Photodetector Based on Metal Oxide Electron/Hole Extraction Layer Formation Conditions

**DOI:** 10.3390/nano15141107

**Published:** 2025-07-16

**Authors:** JinBeom Kwon, Yuntae Ha, Suji Choi, Donggeon Jung

**Affiliations:** 1 Advanced Mobility System Group, Korea Institute of Industrial Technology (KITECH), Daegu 42994, Republic of Korea; jinbum0301@kitech.re.kr (J.K.); hayt223@kitech.re.kr (Y.H.); suji983@kitech.re.kr (S.C.); 2 School of Electronic and Electrical Engineering, Kyungpook National University, Daegu 41566, Republic of Korea

**Keywords:** quantum dots, PbS, SWIR, LiDAR sensor

## Abstract

Recently, with the development of automation technology in various fields, much research has been conducted on infrared photodetectors, which are the core technology of LiDAR sensors. However, most infrared photodetectors are expensive because they use compound semiconductors based on epitaxial processes, and they have low safety because they use the near-infrared (NIR) region that can damage the retina. Therefore, they are difficult to apply to automation technologies such as automobiles and factories where humans can be constantly exposed. In contrast, short-wavelength infrared photodetectors based on PbS QDs are actively being developed because they can absorb infrared rays in the eye-safe region by controlling the particle size of QDs and can be easily and inexpensively manufactured through a solution process. However, PbS QDs-based SWIR photodetectors have low chemical stability due to the electron/hole extraction layer processed by the solution process, making it difficult to manufacture them in the form of patterning and arrays. In this study, bulk NiO and ZnO were deposited by sputtering to achieve uniformity and patterning of thin films, and the performance of PbS QDs-based photodetectors was improved by optimizing the thickness and annealing conditions of the thin films. The fabricated photodetector achieved a high response characteristic of 114.3% through optimized band gap and improved transmittance characteristics.

## 1. Introduction

Recently, as autonomous driving and smart factories, etc. are actively developing automation technologies, the demand for LiDAR sensors is rapidly increasing. LiDAR sensors are a technology that scans the surrounding environment in real time and accurately identifies objects, vehicles, and people through 3D mapping, and are considered the core of automation technology. LiDAR sensors consist of infrared photodetectors, infrared lasers, drive motors, and signal processing units. Among these, infrared photodetectors are essential technologies that detect light reflected from a light source to enable 3D mapping, and are considered the core technology that determines the performance of LiDAR sensors [1,2,3,4,5,6,7,8]. The infrared photodetectors currently applied to LiDAR sensors use InGaAs-based photodetectors that detect the near-infrared (NIR) region, and have high sensitivity and excellent stability [9,10]. However, because it requires a complex process based on the epitaxial growth of silicon and III-V semiconductor compounds, the manufacturing cost is high, and a cooling device is essential due to thermal noise when operating at room temperature. In addition, NIR-based photodetector technology using a wavelength range of 700 to 1000 nm has a major problem in that it can penetrate the human retina and cause damage. Therefore, research on SWIR photodetectors using shortwave infrared has been continuously conducted to improve these limitations. SWIR is an infrared light with a wavelength range of 1000 to 2500 nm, and can provide superior image resolution compared to near-infrared light at night and in bad weather due to its high transmittance and low scattering characteristics. In addition, SWIR in the wavelength band of 1400 nm to 2500 nm is an eye-safe band and does not penetrate the human retina, so it is highly safe. Among various SWIR photodetectors, interest in quantum dot (QD)-based photodetectors is increasing. In particular, lead sulfide (PbS) quantum dots have the advantage of being easy and inexpensive to manufacture because they can be synthesized and manufactured into components through a solution process without a complex semiconductor process [11,12,13,14,15]. In addition, since the wavelength range of 1000–2800 nm can be adjusted by controlling the particle size, photodetectors can be manufactured in a wavelength range that is safe for the eyes. Although PbS QDs have these advantages, PbS QD-based SWIR photodetectors reported to date have limitations in the manufacturing process [16,17,18,19,20]. Most layers except electrodes, such as the electron extraction layer, photoactive layer, and hole extraction layer, are all formed into thin films through a solution process, and these thin films are vulnerable to processes such as photolithography and etching due to their low chemical stability [21,22,23]. Therefore, it is impossible to form a thin film pattern, which makes it difficult to form a sensor array, and there are limits to the application of the LiDAR system. Therefore, in this study, bulk NiO and bulk ZnO were deposited as hole extraction layers and electron extraction layers, respectively, using sputtering and a shadow mask to overcome the existing solution-processed extraction layer that cannot be patterned. In addition, in order to confirm the characteristics according to the thickness of each layer, the sputter deposition time was adjusted to adjust the deposition thickness, and to confirm the characteristics according to annealing conditions, characteristic analysis according to annealing temperature and time was performed. In order to analyze the band gap according to the thickness of ZnO, the optical band gap of the ZnO thin film was measured, and in order to analyze the characteristics according to the thickness and annealing conditions of NiO, the transmittance, grain size, and grain boundaries of the NiO thin film were measured. In addition, to analyze the characteristics of the SWIR photodetector according to the NiO and ZnO thin film conditions, the current and sensitivity were measured according to the light source irradiation. As a result, the fabricated SWIR photodetector has a sensitivity of up to 114.3% with an error rate of 1.14% by optimizing the ZnO deposition conditions to form a quantum well structure and optimizing the NiO deposition conditions to improve the transmittance at 1405 nm IR.

## 2. Experimental Details

### 2.1. Materials

Lead oxide (PbO, 99.99%), sulfur (S, 99.98%), oleic acid (OA, technical grade, 70%), 1-octadecene (1-ODE, 94%), trioctylphosphine (TOP, 90%), and anhydrous toluene and ethanol were purchased from Sigma-Aldrich (Burlington, MA, USA). NiO and ZnO sputter target source were purchased from ITASCO (Seoul, Republic of Korea).

### 2.2. Synthesis of Colloidal PbS QDs

PbS QDs were prepared using similar previously reported methods with modifications. In a typical synthesis, two separate solutions containing 2.4 mmol of PbO and 0.42 mmol of sulfur in 0.265 mL of OA, respectively, were stirred for 30 min at room temperature under Ar gas flow. Then, the PbO-OA mixture was heated to 200 °C for 1 h before cooling to 100 °C under vacuum degassing for 15 min. Then, the prepared S-OA solution and 265 µL of TOP were quickly injected into the reactor at elevated temperature under Ar gas flow. The reaction temperature was maintained at 120 °C for 60 min to grow PbS QDs. Synthesized QDs were purified by adding a toluene and ethanol solution, followed by centrifugation at 5000 rpm for 20 min to separate QDs through precipitation. The supernatant liquid phase was decanted to remove the excess reagent; then, QDs were dispersed in a non-polar toluene solution at the concentration of 20 mg/mL

### 2.3. Device Fabrication

**Figure 1:**
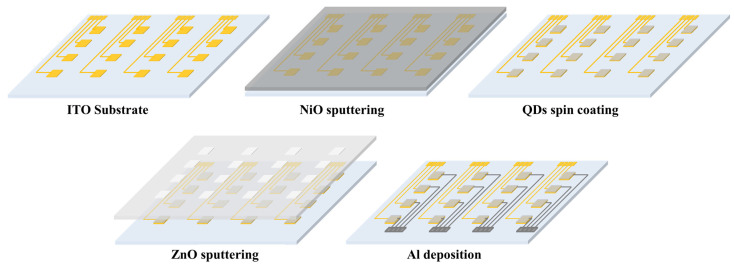
Schematic diagram of SWIR photodetector fabrication.

The proposed SWIR photodetector is fabricated on the glass substrates coated with a patterned indium tin oxide (ITO) anode. The ITO anode had a thickness of approximately 400 Å and a surface resistance of less than 8 Ω. Initially, to remove contamination on the ITO-patterned glass, the glass was cleaned with acetone, methanol, and deionized water and then exposed to UV ozone (AH-1700, AHTECH LTS Co., Ltd., Anyang, Republic of Korea) for 15 min. The NiO hole extraction layer was deposited through a shadow mask using an RF sputter, with an intensity of 200 W and an Ar atmosphere of 15 sccm at a chamber pressure of 20 mTorr. To optimize the thickness, the thickness was adjusted by controlling the deposition time from 2000 to 5000 s. In order to confirm the properties according to the annealing conditions, annealing was performed at a temperature range of room temperature to 400 °C to form a NiO layer, and the annealing time was adjusted from 1 h to 5 h. To form the photoactive layer, the synthesized PbS QDs solution was coated on the substrate by spin-coating method (LT-MS 200, LTS, Anyang, Republic of Korea). According to the optimized spin-coating conditions reported through previous research, it was deposited at 2500 rpm for 30 s, and annealed in a vacuum oven (ov–11, JEIO Tech, Daejeon, Republic of Korea) at a temperature of 110 °C for 30 min to form a PbS QDs thin film, which is a photoactive layer. The ZnO electron extraction layer was also deposited through a shadow mask using an RF sputter, and was deposited in an Ar atmosphere of 15 sccm with a power of 150 W in a chamber with a pressure of 20 mTorr. For optimization according to thickness, the thickness was adjusted and the characteristics were confirmed by adjusting the deposition time under the condition of 500~3000 s. Finally, for cathode deposition, Al was deposited through a shadow mask using thermal evaporation, and was deposited to a thickness of over 200 nm. The size of one sensing cell of the fabricated device was formed as 2 × 2 mm^2^. The schematic of the device fabrication is shown in Figure 1.

### 2.4. Device Measurement System

In order to evaluate the characteristics of the fabricated SWIR photodetector, the current change characteristics according to IR light source irradiation were confirmed. All measurements were performed in a dark chamber to eliminate interference from external light. After fixing the fabricated photodetector with a probe tip, measurements were performed by applying voltage through a source meter unit (SMU, B2902A, Keysight, Santa Rosa, CA, USA). An IR light source (SLS 202L/M, Thorlabs, Newton, NJ, USA) was installed at the top of the chamber, and the dark current (I_dark_), which is the current value when the light is not irradiated, and the photocurrent (I_light_), which is the current value when the light is irradiated, were measured and the sensitivity was analyzed.

## 3. Results

### 3.1. Characteristics of the Synthesized PbS QDs

**Figure 2:**
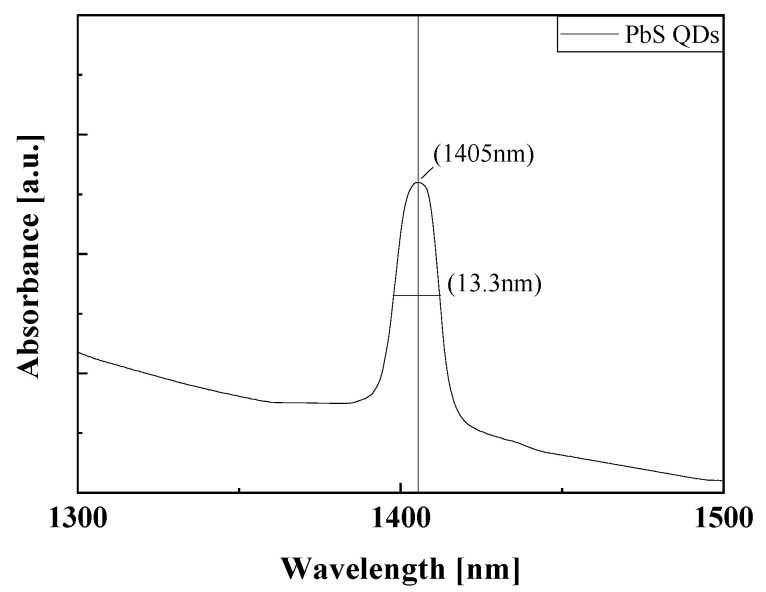
Absorption spectra of the synthesized PbS QDs.

**Figure 3:**
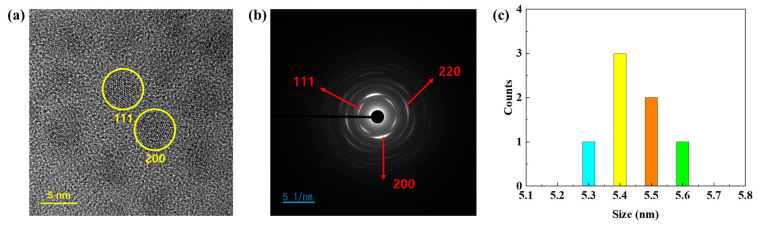
(**a**) TEM image, (**b**) SAED, and (**c**) size distribution of the synthesized PbS QDs.

The synthesized QDs were analyzed by absorbance and transmission electron microscopy (TEM), and the properties and success rate of synthesis were evaluated. As shown in Figure 2, the synthesized QDs were analyzed by absorbance, X-ray diffraction (XRD), and transmission electron microscopy (TEM), and the properties and success rate of synthesis were evaluated. The absorbance spectrum of the synthesized PbS QDs showed an absorption wavelength peak of 1405 nm with a narrow FWHM of 13.3 ± 1% nm. It exhibits high absorbance with high selectivity in the eye-safe infrared region. Quantum confinement changes the wavelength band of quantum dots depending on the particle size. Based on quantum confinement, if the particle size is smaller than the Bohr radius, the instability increases and the band gap of the particle increases as the particle size decreases. According to equation (1), the band gap is inversely proportional to the wavelength band. Since the band gap is inversely proportional to particle size, the wavelength band of PbS QD is directly proportional to its size. Additionally, as confirmed in Figure 3a,c, the particle size of the synthesized PbS QDs was measured to be an average of 5.44 nm through TEM images. As shown in Figure 3b, the lattice space and selected-field electron diffraction (SAED) of PbS QDs were analyzed through TEM images to determine the lattice space and SAED of (111), (220), and (200). These results support that the synthesis of quantum dots was successful [15].

### 3.2. Performance of the SWIR Photodetector

#### 3.2.1. Characteristics According to ZnO Thin Film Thickness

**Figure 4:**
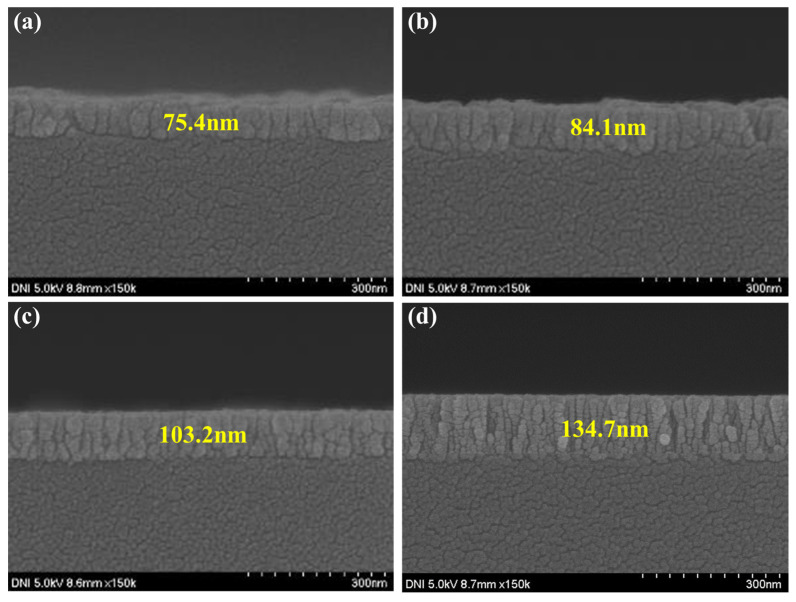
The thickness of the deposited ZnO film: (**a**) 500 s, (**b**) 1000 s, (**c**) 2000 s, and (**d**) 3000 s.

As shown in Figure 4, SEM measurements were performed to check the thickness of the ZnO thin film according to deposition time. As a result, depending on the deposition time of 500, 1000, 2000, and 3000 s, the thin film thickness was confirmed to be 51.6, 73.4, 116, and 226 nm, respectively. In order to confirm the optical band gap according to the ZnO thin film thickness, the absorbance of ZnO according to the thin film thickness was measured and calculated using the tauc plot equation [24].

E = hυ/λ = 1240/wavelength(1)

(αhυ)^2^ = (2.303 × absorbance × E)^2^ (2)

**Figure 5:**
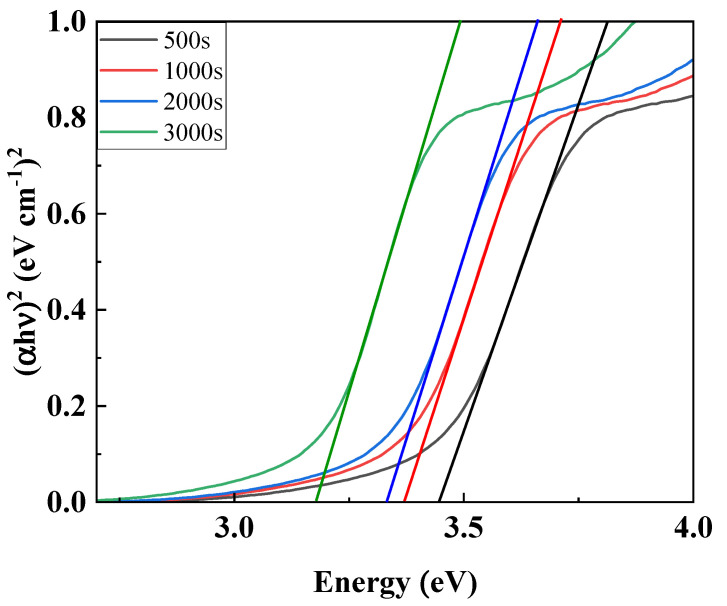
The band gap plot of the deposited ZnO thin films.

**Figure 6:**
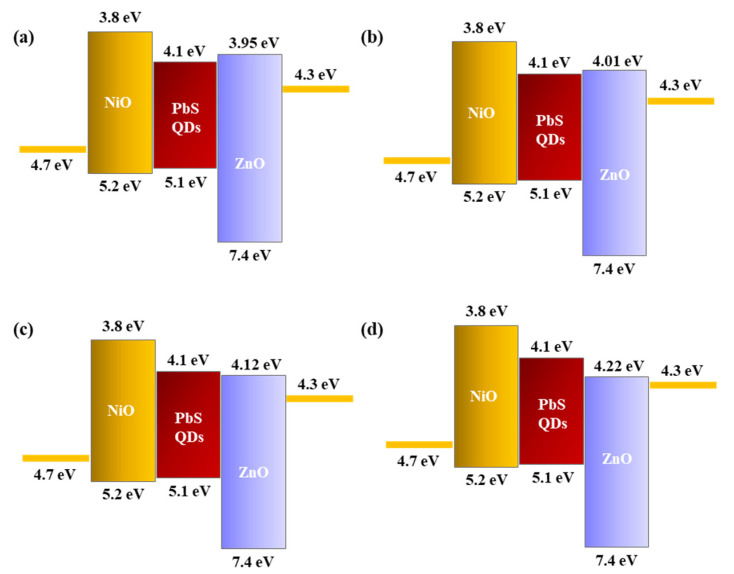
(**a**–**d**) Energy band diagram according to the band gap of ZnO thin films.

Based on the measured absorbance, calculate the energy using equation (1) and set the X-axis, and use Equation (2) to calculate (αhυ)^2^ and set the Y-axis. Next, when the normal line of the slope of the graph is drawn, the intersection with the X-axis can be confirmed, which becomes the optical band gap. As a result, as can be seen in Figure 5 and Table 1, it can be seen that the optical band gap of ZnO decreases as the thickness of the ZnO thin film increases [23,24]. The SWIR photodetector proposed in this study is based on the principle of the photoconductivity effect. When a voltage is applied to the fabricated device and a light source is irradiated, electron–hole pairs are formed in the photoactive layer, PbS QDs, which enhances the overall conductivity, and thus the photocurrent appears as an increased current value. Therefore, the change in the band gap affects the recombination of exciton and electron hole mobility generated in the photoactive layer of the photodetector, which is directly related to the dark current and current change value of the photodetector. If the band gap of ZnO is too large, the overall resistance of the device increases and the dark current decreases, but the electrons generated in the photoactive layer cannot move to the electrode, so the current change decreases and the sensitivity decreases. On the other hand, if the band gap is too small, the overall resistance of the device decreases and the dark current increases, so the sensitivity decreases, and the reaction speed decreases because saturation does not occur when irradiated with a light source due to the recombination of excitons generated in the photoactive layer. Therefore, it is important to form a quantum well structure that forms a well shape based on the photoactive layer with an appropriate level of band gap. The quantum well structure has the characteristic of a low dark current, and can improve the response speed by preventing the recombination of electrons and holes due to the barrier role. Also, if the barrier of the quantum well structure is too high, the excitons generated by the photoreaction can be trapped, so it is important to control the appropriate level [25,26,27,28,29,30,31]. As shown in Figure 6, when confirmed with a band diagram schematic, it was confirmed that the quantum well structure was not formed due to the small band gap at 2000 s and 3000 s, and that the quantum well structure was formed at 500 s and 1000 s [32,33].

**Figure 7:**
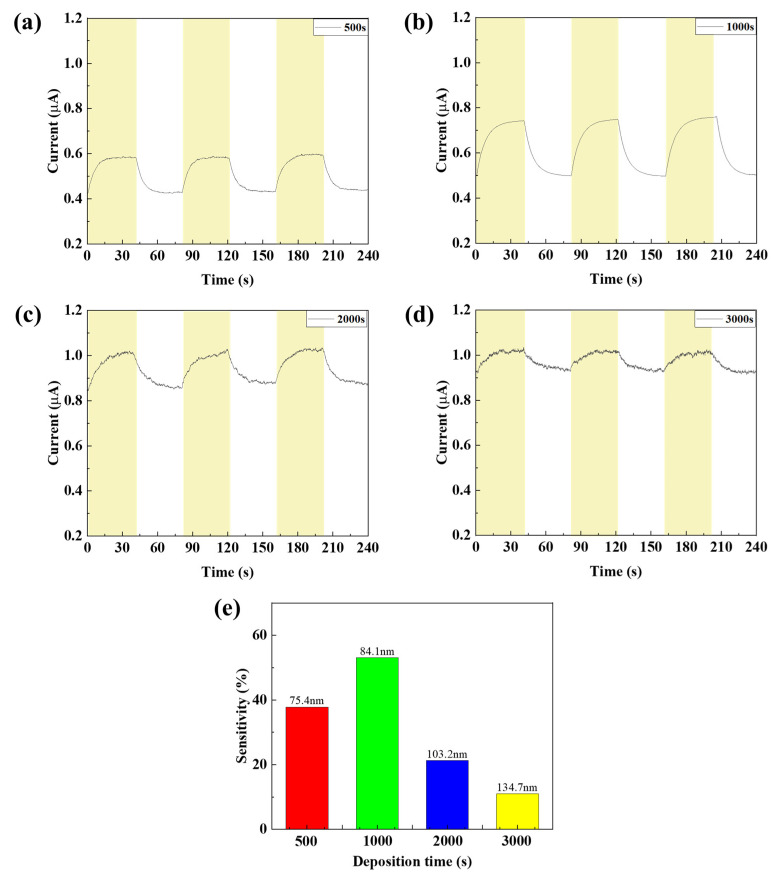
(**a**–**d**) The current characteristics and (**e**) sensitivity of the fabricated SWIR photodetector according to the ZnO thin film thickness.

As a result of measuring the current characteristics of the SWIR photodetector according to the IR light source irradiation according to the ZnO film thickness, different characteristics were confirmed according to the change in the band gap, as shown in Figure 7. As the thickness of the ZnO film increased to 2000 and 3000 s, the overall resistance decreased due to the decrease in the band gap, which confirmed that the dark current increased. In addition, it was confirmed that the current change decreased because the quantum well was not formed, which increased the exciton recombination. In the 1000 s condition, the quantum well was formed due to the increased band gap, showing low dark current characteristics, and it was confirmed that the current change increased because the recombination of excitons generated in the photoactive layer was prevented due to the barrier. In the 500 s condition, the lowest dark current was shown due to the largest band gap, but it was confirmed that the current change decreased because the quantum well was formed so large that the electrons could not be smoothly discharged to the electrode. As a result of calculating the sensitivity according to the current change value, the sensitivity was confirmed to be 53.1%, which was up to 4.8 times higher, under the 1000 s ZnO deposition condition.

#### 3.2.2. Characteristics According to NiO Thin Film Thickness

**Figure 8:**
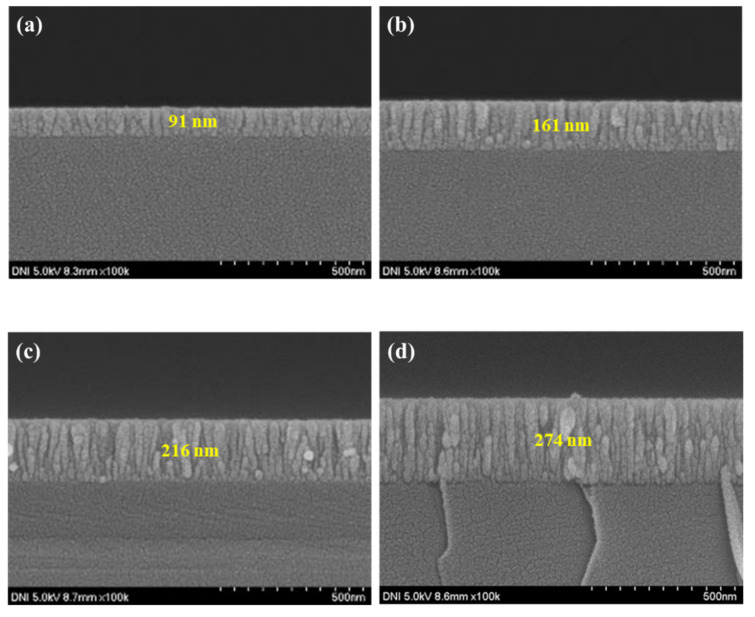
The thickness of the deposited NiO thin film: (**a**) 2000 s, (**b**) 3000 s, (**c**) 4000 s, and (**d**) 5000 s.

**Figure 9:**
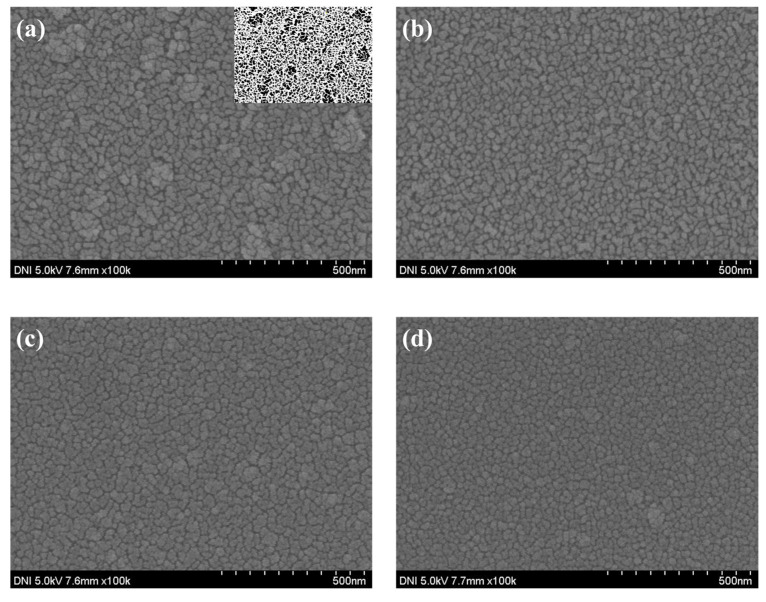
The surface image of the deposited NiO thin film: (**a**) 2000 s, (**b**) 3000 s, (**c**) 4000 s, and (**d**) 5000 s.

**Figure 10:**
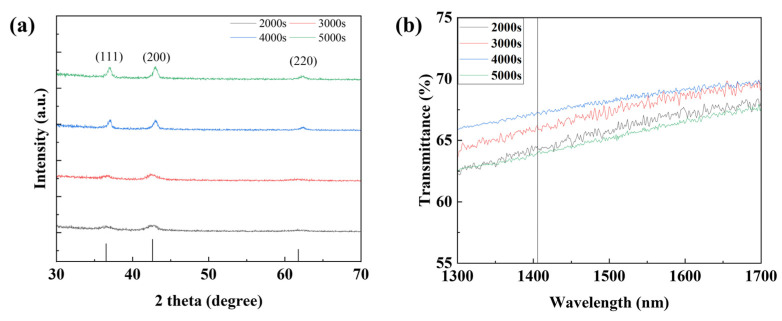
(**a**) The XRD analysis and (**b**) transmittance property of the NiO thin film according to the deposition time.

**Figure 11:**
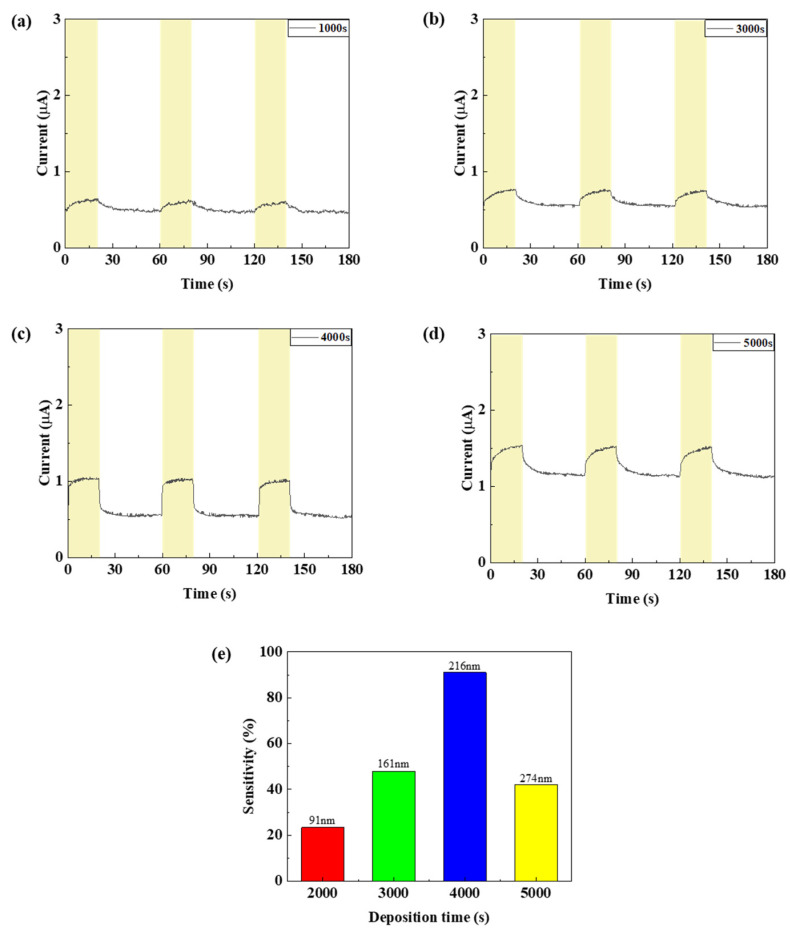
(**a**–**d**) The current characteristics and (**e**) sensitivity of the fabricated SWI R photodetector according to the NiO thin film thickness.

To confirm the device characteristics according to the NiO deposition time, the thickness and particle size of the NiO thin film were measured through SEM measurement, and the average particle size was obtained using the ImageJ program version 1.54h (NIH% LOCI). As shown in Figure 8 and Figure 9, the thickness of the NiO thin film according to the deposition time of 1500, 3000, 4000, and 5000 s was confirmed to be 91, 161, 216, and 274 nm, respectively, and the grain size according to the thin film thickness was confirmed to be 34.114, 39.581, 40.745, and 44.38 nm. In addition, transmittance was measured using UV-Vis-IR (Cary-5000) to confirm the transmittance of the thin film depending on the thickness. As shown in Figure 10b, it was confirmed that the transmittance in the 1405 nm wavelength band targeted in this experiment increased as the thickness increased up to the deposition time of 4000 s. This is because the uniformity of the thin film is improved and at the same time, light scattering is reduced due to an increase in grain size and a decrease in grain boundaries [34,35]. However, it was confirmed that under deposition conditions of more than 5000 s, the excessive thin film interferes with the transmission of infrared rays and actually reduces the transmittance. To analyze the increase in grain size according to NiO thin film thickness, XRD analysis was performed. As shown in Figure 10a, it was confirmed that the peaks at (111) and (200), which indicate grain growth, increased as the thickness increased. In addition, as a result of calculating the grain size using Scherrer’s equation, it was confirmed that the grain size increased to a similar value as confirmed in the SEM image. Table 2 summarizes the thickness, transmittance, and grain size according to NiO deposition time. Additionally, by the same mechanism as the ZnO thin film, the optical band gap increases as the thickness increases. If the band gap is too low, saturation does not occur due to the recombination of excitons generated in the photoactive layer, and the sensitivity decreases due to low current variances. As a result of measuring current characteristics, it was confirmed that as deposition time increased, the dark current decreased due to an increase in optical band gap. In addition, it was confirmed that the amount of current variance increased due to an increase in the intensity of infrared irradiation to the photoactive layer due to an increase in the transmittance of the NiO thin film. As a result of calculating the response, as can be seen in Figure 11, it was confirmed that the sensitivity was 91%, up to 3.9 times higher, with the lowest dark current and highest current variance under the condition of 4000 s. On the other hand, it was confirmed that the current variance and sensitivity decreased from the 5000 s condition as the transmittance decreased.

#### 3.2.3. Characteristics According to NiO Thin Film Annealing Conditions

**Figure 12:**
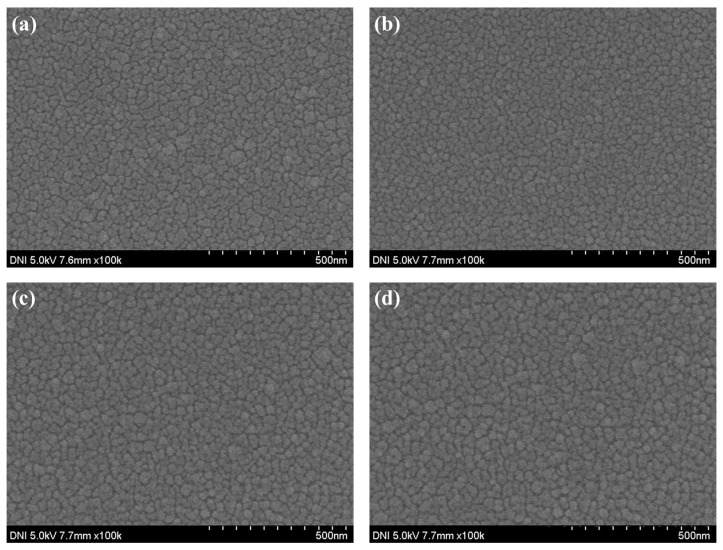
The surface image of the deposited NiO thin film according to the annealing temperature: (**a**) R.T., (**b**) 150 °C, (**c**) 300 °C, and (**d**) 400 °C.

**Figure 13:**
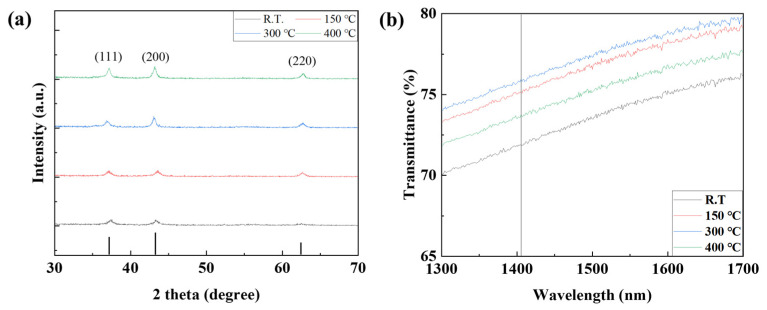
(**a**) The XRD analysis and (**b**) transmittance properties of the NiO thin film according to the NiO annealing temperature.

**Figure 14:**
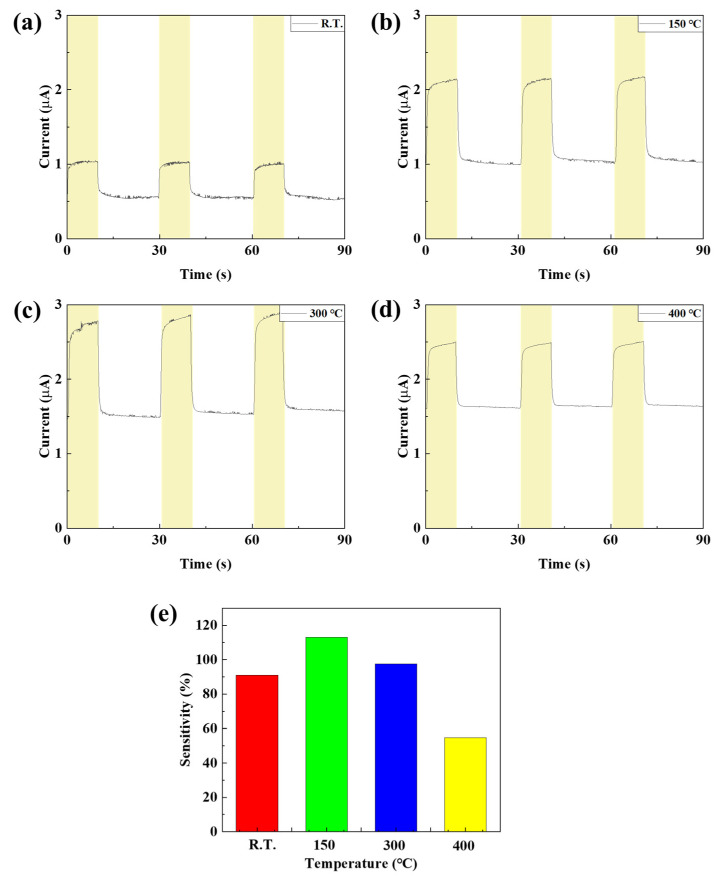
(**a**–**d**) The current characteristics and (**e**) sensitivity of the fabricated SWIR photodetector according to the NiO annealing temperature.

In order to confirm the device characteristics according to the annealing conditions of the NIO thin film, two steps of temperature and time were performed using the NiO thin film deposited with the previously optimized deposition time of 4000 s. To confirm the properties according to annealing temperature conditions, a photodetector was manufactured by annealing the NiO thin film at room temperature, 150, 300, and 400 °C, and the properties were evaluated. First, the grain size and grain boundary characteristics of the NiO thin film according to annealing temperature were analyzed through SEM image measurement. As shown in Figure 12 and Table 3, it was confirmed that the grain size increased significantly as the temperature increased, and in particular, the grain boundary significantly decreased from 300 °C. It was confirmed that the grain boundary was further reduced at 400 °C, but damage and discoloration were confirmed at a temperature of 400 °C due to the limitations of the ITO electrode deposited on the bottom of the NiO thin film. Therefore, annealing was not performed at temperatures above 400 °C. When a metal oxide thin film is heated, the grain size increases due to grain recrystallization and growth, and the grain boundary decreases accordingly. Grain boundary refers to the gap between grains, and can act as a defect, trapping electrons during charge transfer. This not only reduces the reaction speed and recovery speed of the photodetector, but also causes scattering of light, reducing light transmittance and reducing the responsiveness of the photodetector. In addition, a decrease in grain boundaries reduces resistance due to a decrease in defects, but in a photodetector, low resistance means a high dark current, so optimization through appropriate grain size control is essential [36,37]. XRD analysis was performed to confirm crystallinity according to annealing temperature. As shown in Figure 13, compared to room temperature, the peak at (220) became clear from 150 °C and it was confirmed that the crystallinity of NiO was improved. It was confirmed that from 300 °C, the peaks of (111) and (200) increased, the grain size of the NiO thin film increased, and the grain boundary decreased. As a result of measuring the absorption characteristics to analyze the transmittance of the NiO thin film according to temperature, the highest transmittance characteristics were confirmed at 300 °C. At 400 °C, a decreased transmittance characteristic was observed. This is because ITO has a limit to damage above 400 °C, and it was confirmed that the transmittance was reduced by ITO. Figure 14 shows the current change rate and response to the light source, and a large current change rate was confirmed due to the high transmittance of NiO under the annealing conditions of 150 °C and 300 °C, and the highest response rate was confirmed with low dark current characteristics at 150 °C.

**Figure 15:**
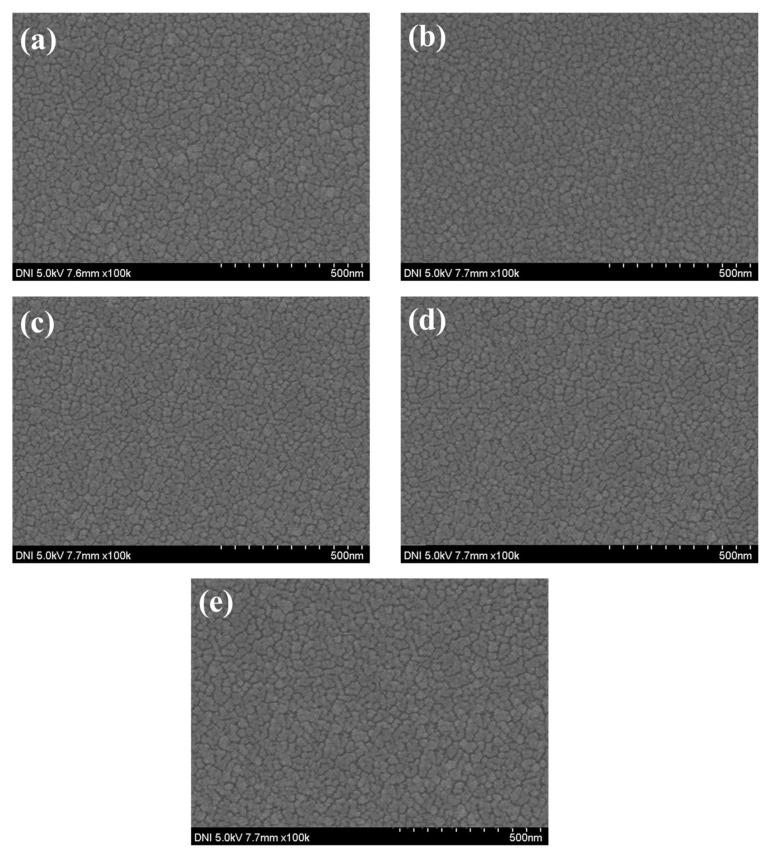
The surface image of the deposited NiO thin film according to the annealing time: (**a**) R.T., (**b**) 1 h, (**c**) 2 h, (**d**) 3 h, and (**e**) 4 h.

**Figure 16:**
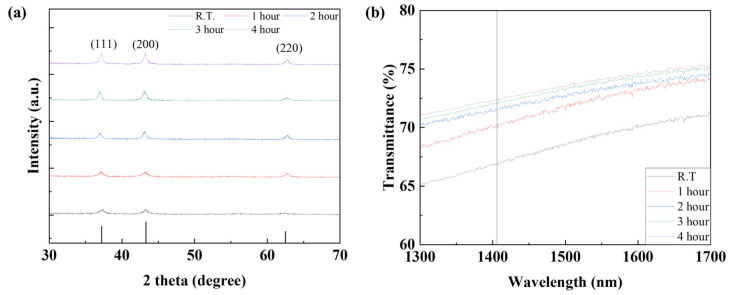
(**a**) The XRD analysis and (**b**) transmittance properties of the NiO thin film according to the NiO annealing time.

**Figure 17:**
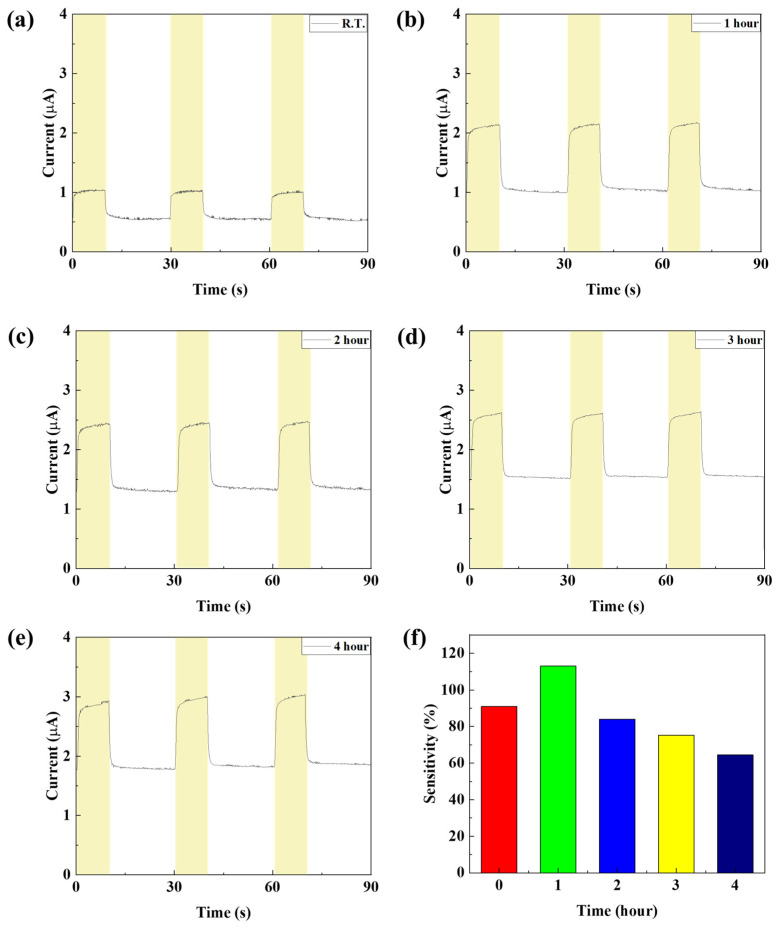
(**a**–**e**) The current characteristics and (**f**) sensitivity of the fabricated SWIR photodetector according to the NiO annealing time.

In order to confirm the device characteristics according to the annealing time of the NiO thin film, the device was annealed at a temperature of 150 °C for 1, 2, 3, and 4 h and the device characteristics were analyzed. To confirm the grain characteristics of the NiO thin film according to the annealing time, SEM image measurement and analysis of the thin film surface were performed. As shown in Table 4 and Figure 15, it was confirmed that when annealed for 1 h, recrystallization of the thin film occurred and the uniformity of the thin film was improved. In addition, it was confirmed that as the annealing time increased, the grain size slightly increased. To analyze the crystal properties of the thin film confirmed by SEM, XRD measurement was performed. As shown in Figure 16, when annealed for 1 h, it was confirmed that the (220) peak, which represents the peak of the NiO thin film, became clear due to recrystallization, and it was confirmed that the (111) and (220) peaks increased as time increased. Although this is a smaller value compared to the result resulting from an increase in annealing temperature, it can be proven that the grain size of the thin film increases as the annealing time increases even at a temperature of 150 °C. The infrared transmittance of the NiO thin film according to the annealing time was also measured, and it was confirmed that the transmittance increased as the annealing time increased. This can be confirmed through improved grain size confirmed through XRD and SEM image analysis, and at the same time, the grain boundary has been reduced, reducing light scattering and increasing transmittance. In addition, unlike the transmittance that decreased due to damage to the thin film under the condition of 400 °C, it was confirmed that the transmittance increased without damage to the thin film as time increased and became saturated under the relatively low temperature condition of 150 °C. As a result of measuring the current characteristics of the photodetector fabricated according to the annealing time conditions, it was confirmed that the dark current increased due to the decrease in thin film resistance due to the increase in grain size and decrease in grain boundary, and the amount of current change also increased as the transmittance improved. As shown in Figure 17, the highest sensitivity was observed at 114.3%, up to 1.63 times higher, under the 1 h annealing condition with a relatively low dark current and high current variance. Under longer annealing time conditions, the current variance further increased due to the increased transmittance of the NiO thin film, but the dark current increased due to the increased conductivity caused by the increased grain size and the decreased grain boundary, which resulted in a decrease in sensitivity.

**Figure 18:**
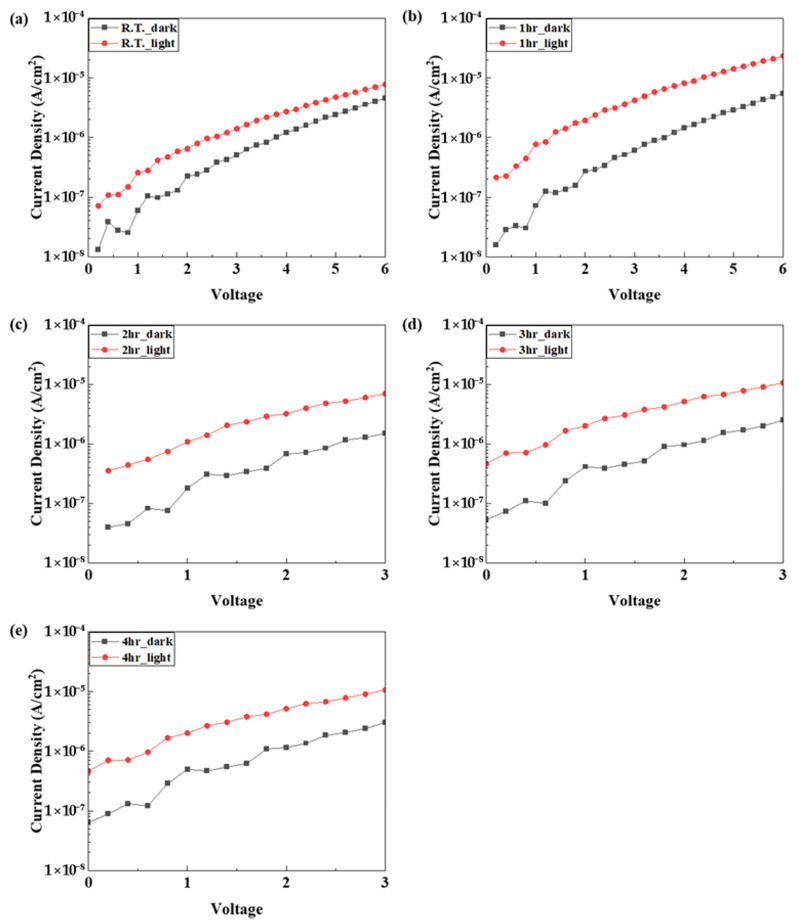
(**a**–**e**) The C-V characteristics of the fabricated SWIR photodetector according to the NiO annealing time.

To verify the sensitivity of the photodetector according to the applied voltage, the device was swept from 0 to 6 V according to the annealing time and the dark current and light current were measured. In order to compare the on/off ratio of the dark current and the light current, the measured values were expressed in a log scale. As shown in Figure 18, the measurement results show that the sample annealed for 1 h showed the largest on/off ratio at 2 V, and the optimized device showed the best performance when the operating voltage was 2 V.

**Figure 19:**
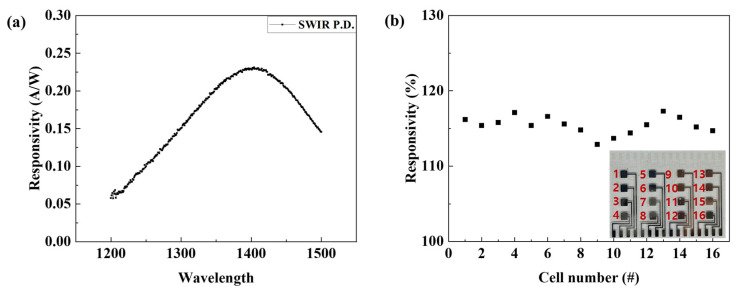
(**a**) Responsivity, (**b**) sensitivity of all cells according to wavelength band of fabricated SWIR photodetector and cell number of actual device image.

The sensitivity of the fabricated SWIR photodetector according to the wavelength band was measured. As a result of measuring the sensitivity in the wavelength band from 1200 to 1500 nm, the highest sensitivity characteristic of 0.23 A/W was confirmed in the 1405 nm wavelength band, as shown in Figure 19a. This confirms that the fabricated SWIR photodetector has low sensitivity to infrared other than 1405 nm and can have selectivity to the 1405 nm infrared. In addition, the sensitivity of each cell of the patterned sensor was confirmed. The fabricated device consisted of a total of 16 cells, and the current change according to the infrared light source was measured from cell 1 to 16, and the response was calculated. As shown in Figure 19b, the fabricated device was confirmed to exhibit high reliability with an average sensitivity of 114.3% and an error rate of 1.14%. Table 5 summarizes the device structure and performance of typical QD-based SWIR detectors. Compared with recently reported QDs-based SWIR photodetectors, it was confirmed to have selective detection in the eye-safety band and high responsivity.

## 4. Conclusions

Herein, we developed a highly sensitive eye-safety SWIR photodetector based on PbS QDs. The synthesized PbS QDs have a peak absorption wavelength of 1405 nm and a half width of 12.3 nm, which can detect infrared in the eye-safety band with high selectivity. After that, the device was fabricated by sequentially depositing NiO, PbS QDs, ZnO, and Al electrodes on the patterned ITO glass. The fabricated sensor had high transmittance due to the optimized thickness annealing temperature and time of bulk NiO, and could detect infrared rays with high sensitivity due to the optimized band gap of bulk ZnO. The sensor fabricated by optimizing the band gap of ZnO forms a quantum well structure, minimizing recombination and reducing the dark current, resulting in high response and response speed. By optimizing the thickness and annealing conditions of NiO, the scattering of infrared rays is reduced and the transmittance is improved, resulting in high response. As a result, the fabricated sensor has secured a high response characteristic of 114.3% for eye-safe infrared rays in the 1405 nm wavelength band, and has endless possibilities for array-type production and application to image sensors through NiO and ZnO pattern deposition via sputtering.

## Figures and Tables

**Table 1:** The thickness and band gap according to the ZnO deposition conditions.

Deposition Time (s)	Thickness (nm)	Band Gap (eV)
500	75.4	3.45
1000	84.1	3.39
2000	103.2	3.28
3000	134.7	3.18

**Table 2:** The thickness, transmittance, and grain size according to the NiO deposition conditions.

Deposition Time (s)	Thickness (nm)	Transmittance (%)	Grain Size (nm)
2000	91	64.3	34.114
3000	161	66.1	38.581
4000	216	67.3	40.745
5000	274	64	44.38

**Table 3:** The thickness, transmittance, and grain size according to the NiO annealing temperature.

Temperature (°C)	Transmittance (%)	Grain Size (nm)
R.T.	71.3	40.745
150	74.1	44.872
300	75.8	47.937
400	73.6	49.264

**Table 4:** The thickness, transmittance, and grain size according to the NiO annealing time.

Temperature (°C)	Transmittance (%)	Grain Size (nm)
R.T.	71.3	40.745
1	74.1	44.872
2	74.5	45.127
3	75.0	49.724
4	75.4	51.548

**Table 5:** Summary and comparison of performance of QDs-based photodetectors.

Material	Device Structure	Response Band (nm)	Responsivity (A/W)	Ref.
PbS	Si/ZnO/PbS	1310	0.22	[38]
PbSe	InSnZnO/PbSe	2100	3.91 × 10^−3^	[39]
In(As,P)	ITO/NiO/In(As,P)/TiO_2_/Al	1400	7 × 10^−3^	[40]
HgTe	Bi2S_3_/HgTe/Ag:HgTe	2200	0.29	[41]
PbSe	PbSe	400−2600	0.32	[42]
PbS	ZnO/PbS	1310	0.47	[43]
Cu_2_SnS_3_	ITO/Cu_2_SnS_3_/Ag	1550	0.9 × 10^−3^	[44]
Ag_2_Te	Ag_2_Te/AgNiS_2_/SnO_2_	350−1600	0.1	[45]
PbS	ITO/NiO/PbS/ZnO/Al	1405	0.23	This work

## Data Availability

The raw data supporting the conclusions of this article will be made available by the authors on request.
